# Dose rate in the highest irradiation area of the rectum correlates with late rectal complications in patients treated with high-dose-rate computed tomography-based image-guided brachytherapy for cervical cancer

**DOI:** 10.1093/jrr/rrab023

**Published:** 2021-04-19

**Authors:** Fumiaki Isohashi, Yuichi Akino, Yuri Matsumoto, Osamu Suzuki, Yuji Seo, Keisuke Tamari, Iori Sumida, Kenjiro Sawada, Yutaka Ueda, Eiji Kobayashi, Takuji Tomimatsu, Erina Nakanishi, Takahisa Nishi, Tadashi Kimura, Kazuhiko Ogawa

**Affiliations:** Department of Radiation Oncology, Osaka University Graduate School of Medicine, Osaka, 565-0871, Japan; Department of Radiation Oncology, Osaka University Graduate School of Medicine, Osaka, 565-0871, Japan; Department of Gynecology and Obstetrics, Osaka University Graduate School of Medicine, Osaka, 565-0871, Japan; Department of Radiation Oncology, Osaka University Graduate School of Medicine, Osaka, 565-0871, Japan; Department of Radiation Oncology, Osaka University Graduate School of Medicine, Osaka, 565-0871, Japan; Department of Radiation Oncology, Osaka University Graduate School of Medicine, Osaka, 565-0871, Japan; Department of Radiation Oncology, Osaka University Graduate School of Medicine, Osaka, 565-0871, Japan; Department of Gynecology and Obstetrics, Osaka University Graduate School of Medicine, Osaka, 565-0871, Japan; Department of Gynecology and Obstetrics, Osaka University Graduate School of Medicine, Osaka, 565-0871, Japan; Department of Gynecology and Obstetrics, Osaka University Graduate School of Medicine, Osaka, 565-0871, Japan; Department of Gynecology and Obstetrics, Osaka University Graduate School of Medicine, Osaka, 565-0871, Japan; Department of Radiation Oncology, Osaka University Graduate School of Medicine, Osaka, 565-0871, Japan; Department of Radiation Oncology, Osaka University Graduate School of Medicine, Osaka, 565-0871, Japan; Department of Radiation Oncology, Osaka International Cancer Institute, Osaka, 541-8567, Japan; Department of Gynecology and Obstetrics, Osaka University Graduate School of Medicine, Osaka, 565-0871, Japan; Department of Radiation Oncology, Osaka University Graduate School of Medicine, Osaka, 565-0871, Japan

**Keywords:** brachytherapy, complications, intracavity radiotherapy, rectum, uterine cervical neoplasm

## Abstract

The purpose of this study was to evaluate the effect of dose rate to the rectum on late rectal complications in patients treated with computed tomography (CT)-based image-guided brachytherapy (IGBT) for cervical cancer. The subjects were 142 patients with cervical cancer who underwent Ir-192 high-dose-rate (HDR)-IGBT between March 2012 and January 2018. The dose rate to the rectum was calculated using in-house software. The minimum, mean and maximum effective dose rate (EDR) was calculated for voxels of the rectal volume covered by cumulative doses >D_0.1cc_, >D_2cc_, and > D_5cc_. The average EDR of three to four brachytherapy sessions was calculated (EDR for patients; EDR_p_). The total dose of the rectum was calculated as the biologically equivalent dose in 2-Gy fractions (EQD_2_). The associations between EDR_p_ for D_0.1cc_, D_2cc_, and D_5cc_ and the respective rectal EQD_2_ values with late rectal complications were then analyzed. The median follow-up period was 40 months. Patients with rectal complications of ≥Grade 1 received a significantly higher mean EDR_p_ for D_0.1cc–5cc_ and had a greater EQD_2_ for D_0.1cc–5cc_. Multivariate analysis was performed using the mean EDR_p_ for D_2cc_, EQD_2_ for D_2cc_, heavy smoking and BMI. Of these four variables, mean EDR_p_ for D_2cc_ (HR = 3.38, p = 0.004) and EQD_2_ for D_2cc_ (HR = 2.59, p = 0.045) emerged as independent predictors for late rectal complications. In conclusion, mean EDR_p_ and EQD_2_ were associated with late rectal complications in patients treated with HDR CT-based IGBT for cervical cancer.

## INTRODUCTION

Brachytherapy has a key role as radiotherapy in radical treatment for cervical cancer [1]. The practice of this treatment has recently changed globally, with increased use of high-dose-rate (HDR) brachytherapy compared to low-dose-rate (LDR) brachytherapy. Treatment planning for brachytherapy has also changed from use of conventional 2-dimensional (2D) radiographs with a dose prescription at standard points to 3-dimensional (3D)-image-guided brachytherapy (IGBT) [2–4].

Decreased toxicities and improved oncologic outcomes of 3D-IGBT have been reported compared with a 2D approach [5], but rectal complications are still a major concern for patients with cervical cancer who are treated with 3D-IGBT. A prospective multicenter study, including patients who received 3D-IGBT for cervical cancer, found an incidence of rectal treatment-related events of 27.6% [6]. After 3D-IGBT, the most significant factor affecting late rectal complications is the total radiation dose, based on the combined doses of external beam radiation therapy (EBRT) and brachytherapy. The total radiation dose can be calculated as the biologically equivalent dose in a 2-Gy fraction (EQD_2_) using a linear quadratic model. A significant correlation has been established between late rectal complications and EQD_2_ using a minimal dose administered to a small volume (0.1–2 cc) in the highest irradiation area [6–8].

The correlation of the source strength of HDR Ir-192 and late rectal complications in patients treated with intracavitary brachytherapy (ICBT) for cervical cancer is uncertain. We previously found that late rectal complications were affected by the source strength of Ir-192, as well as by the rectal dose [9, 10], but other studies have shown no relationship of complications with the source strength [11, 12]. These discrepant results might be due to institutional variations in fractionation schemes, applicators used, and dwell position strategies. Our previous studies also had limitations, including a definition of the rectal point dose that was not based on the International Commission on Radiation Units and Measurements point [9] and use of a computed tomography (CT) scan with insertion of the applicator only for the first brachytherapy session [10]. To address these issues, we considered it necessary to measure the rectal dose rate directly, instead of using the source strength of Ir-192. We have used CT-based 3D-IGBT in all brachytherapy sessions since March 2012 [13], and we have developed software to measure the dose rate directly. In this study, we use this approach to analyze the correlation of the dose rate with late rectal complications after HDR CT-based 3D-IGBT.

## MATERIALS AND METHODS

### Patients

This study was performed as a retrospective chart review and was approved by our institutional review board. Between March 2012 and January 2018, a total of 185 patients with histologically-proven cervical cancer were treated using EBRT and brachytherapy at our hospital. Forty-three patients were excluded from the study: seven who underwent template-based interstitial brachytherapy, 10 who received ICBT less than three times, 10 with local recurrence within one year, and 16 who died or were lost to follow-up within one year. Finally, we analyzed 142 patients who were treated with ICBT three to four times and were followed-up for more than one year.

### Treatment

EBRT and HDR-ICBT were performed as previously described [13]. The initial 30–40 Gy was delivered with a 4-field box; subsequently, pelvic irradiation with a 4-cm wide central shield (CS) was performed. After adequate tumor regression, HDR-ICBT was performed once a week during EBRT with a CS. ICBT was administered in four fractions for patients who received EBRT of 30 Gy, and in three fractions for those who received 40 Gy. Ir-192 was used as the source for HDR-ICBT. No patient was treated with a combination of ICBT applicator and interstitial needles. A CT scan with the applicators inserted was obtained before delivery of each fraction for planning purposes. The high-risk clinical target volume (HR-CTV) and organs at risk (OARs), including the rectum, bladder, sigmoid colon, and small intestine, were contoured on CT according to several guidelines [14–16] and using a treatment planning system (TPS) (Oncentra®; Elekta Inc.).

Our standard loading of dwell positions changed after October 2013. Briefly, we narrowed the dwell position space and increased the number of dwell positions to make it easier to optimize the dose distribution. A summary of this change is shown in Supplementary Table S1. After standard loading of the source dwell positions and weighting with a fraction dose at point A in all patients, dwell times were manually modified using graphical optimization to maximize coverage of the HR-CTV while reducing the dose to the OARs. EQD_2_ was calculated as the combined dose of ICBT and EBRT (excluding fractions with a CS). A value of α/β = 3 was assumed for OARs. Data for the Ir-192 source strength were collected on each day of an ICBT session and the average source strength was calculated over three to four ICBT sessions. Concurrent chemotherapy with platinum-based weekly regimens was used in 104 patients (73%).

### Evaluation of rectal complications

Patients were followed-up by gynecologic and radiation oncologists as outpatients every two months in the first year after treatment, every three months in the second year, every four months in the third year, and every six months after four to five years. Follow-up after five years was done on an individual basis. In all outpatient visits, the patients were interviewed to determine if rectal complications were present, and rectal exams were performed. Rectal complications were assessed according to the Common Terminology Criteria for Adverse Events (CTCAE) v.4.0, including evaluation and grading of the terms proctitis, rectal bleeding, rectal stenosis, and rectal fistula. Late complications were defined as symptoms that persisted and presented beyond three months after radiotherapy.

### Calculation of the effective dose rate

The dose rate to the rectum was calculated using in-house software. For each treatment plan, DICOM RT-Plan, RT-Structure, and CT image data were exported from the TPS. The RT-Plan file contains the 3D coordinates and cumulative weights of each control point. From these data, a unit vector of the orientation of the Ir-192 source and the dwell time were calculated at each dwell position. The rectal contour was segmented into voxels at the resolution of the CT images. The CT slice thickness was 1 mm and the voxel size was 0.48 × 0.48 × 1 mm. For the i^th^ voxel, the dose rate of the radiation exposed at the j^th^ dwell position (DR_i,j_) and the exposed dose (D_i_) were calculated using the formalism of the American Association of Physicists in Medicine Task group No. 43 update 1 (TG-43 U1) [17]:

$$ {DR}_{i,j}\left(r,\theta \right)={S}_k\cdot \Lambda \cdot \frac{G_L\left(r,\theta \right)}{G_L\left({r}_0,{\theta}_0\right)}\cdot{g}_L(r)\cdot F\left(r,\theta \right) $$

$$ {D}_i=\sum_{j=1}^n\left({DR}_{i,j}\cdot{T}_j\right) $$

where r and θ are the distance between the i^th^ voxel and j^th^ dwell position and the elevation angle against the unit vector of the source orientation, respectively; *n* and *T*_j_ are the number of dwell positions and the dwell time of the j^th^ dwell position, respectively; and *S_k_*, *Λ*, *G_L_/G_L0_*, *g*_L_, and *F* are the air Karma strength, dose rate constant, geometric factor, radial dose function, and anisotropy function, respectively. S_k_ at the treatment time was derived from the RT-Plan file.

To validate the accuracy of the in-house software, we calculated the dose in the left and right Point A of the 10 treatment plans of three patients. The mean (min–max) difference between the TPS and in-house software calculations were − 0.04% (−0.38 to 0.20%), showing that the software accurately calculated the brachytherapy dose.

**Fig. 1.**
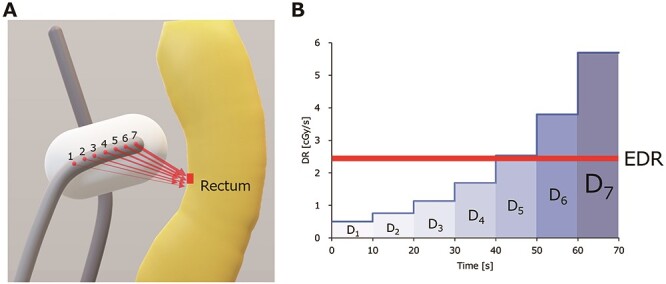
(a) Schematic image of a voxel in the rectum exposed to each dwell position of the ovoid applicator. Thicker arrows represent higher dose rates due to a shorter distance from the dwell position to the voxel. (b) A histogram of the dose rate delivered to the rectal voxel from each dwell position. The dwell time was 10 s for each position. The area of each column calculated as the dose rate multiplied by the dwell time (D_1–7_) represents the cumulative dose delivered from each dwell position. The horizontal dashed line represents the EDR.

Previous studies have investigated the correlation between the Ir-192 source strength and clinical outcomes [9, 10], but the dose rate of photons to critical organs varies among treatments, depending on the distance between the tissue and the dwell positions. Figure 1A shows a schematic image of a voxel of the rectum receiving a dose from seven dwell positions of the ovoid. The voxel receives a dose with the highest dose rate from the dwell position at the tip of the applicator (#7) because this has the shortest distance to the voxel. Figure 1B shows the histogram of the dose rate at one voxel of the rectal tissue. The horizontal axis represents the elapsed time. In this case, the dwell time of each position is 10 s. The vertical axis represents the dose rate that the rectal tissue receives from each dwell position. The area of each column represents the cumulative dose delivered from each dwell position. In this study, an effective dose rate (EDR) was calculated to investigate the impact of the instantaneous dose rate on complications of the rectum, using the following formula:

$$ {EDR}_i=\sum_{j=1}^n\left({w}_{i,j}\cdot{DR}_{i,j}\right)=\sum_{j=1}^n\left(\frac{DR_{i,j}\cdot{T}_j}{Dose_i}\cdot{DR}_{i,j}\right) $$

where EDR_i_ is the dose rate corrected by the contribution of each dwell position to the i^th^ voxel.

**Fig. 2.**
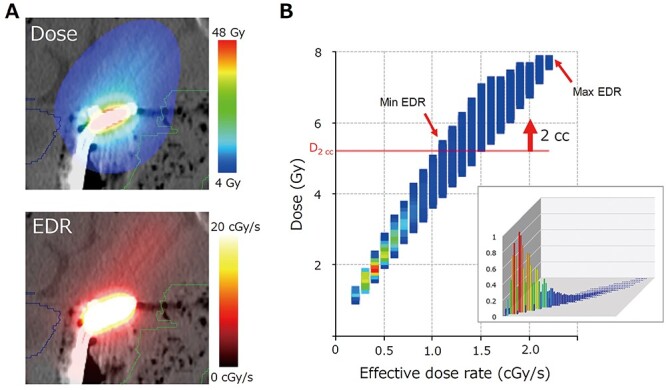
(a) The cumulative dose distribution (upper panel) and EDR (lower panel) in an example case. (b) Typical example of an EDR. The horizontal and vertical axes represent the EDR and the cumulative dose delivered to each voxel, respectively. All voxels were classified with consideration of the EDR with a 0.1 cGy/s step and the cumulative dose with a 10 cGy step. The number of voxels that have an identical dose and EDR are represented by different colors. The data point in red represents that highest number of voxels receiving the identical dose and EDR shown in the chart. The horizontal red line represents the level of D_2cc_, indicating that the total volume of voxels above the line is 2 cm^3^. The minimum and maximum values of EDR > D_2cc_ are indicated by red arrows.

The weight (w*_i, j_*) represents the ratio of the dose delivered from the *j*^th^ dwell position (D*_i,j_)*, shown in Fig. 1b as each column, to the cumulative dose at the i^th^ voxel, shown in Fig. 1b as the total area of the columns. Figure 2a shows the cumulative dose (upper row) and the EDR (lower row). In Fig. 2b, the cumulative dose was plotted against the EDR voxel-by-voxel. The red line represents the minimum dose delivered to 2 cm^3^ of the rectum (D_2cc_). The total volume of the points plotted above this line is 2 cm^3^.

The minimum, mean and maximum EDRs for voxels with the cumulative dose >D_2cc_ were evaluated. The definition of mean EDR for D_2cc_ was the average of all voxel EDRs in the area exposed to a dose above D_2cc._ We also evaluated the EDRs for >D_0.1cc_ and > D_5cc_. For the tandem-ovoid cases, the weight was calculated with consideration of all applicators, including the tandem and two ovoid applicators. This EDR was a single session value and is expressed as the ‘EDR for each session’ (EDR_e_). Since each patient received three to four ICRT sessions, the value per patient was calculated as the sum of EDR_e_ for each session divided by the number of ICRT sessions. This is expressed as the ‘EDR for patients’ (EDR_p_).

### Statistical analysis

Associations between selected parameters and the incidence of Grade 1 or higher late rectal complications were evaluated. The mean EDR_p_ and EQD_2_ for patients with or without late rectal complications were compared by Mann–Whitney *U* test. Relationships between clinical parameters and the incidence of complications were analyzed by Fisher exact test for categorical variables. Receiver operating characteristics (ROC) curve analysis was performed to select thresholds for late rectal complications. Correlations between source strength and EDR_e_, dose and EDR_e_ and dose and source strength for each application were determined by Pearson correlation coefficient analysis. Multivariate analysis using a Cox proportional hazard model was performed using the mean EDR_p_ for D2cc (<98.2 vs ≥98.2), EQD2 for D_2cc_ (<66.7 vs ≥66.7), heavy smoking (Brinkman index < 200 vs ≥200) and BMI (<23.3 vs ≥23.3 kg/m^2^) to identify risk factors associated with late rectal complications. The actuarial incidence of late rectal complications was calculated using the Kaplan–Meier method, with differences between groups compared by Log-rank test. A *P*-value of < .05 or a 95% confidence interval not encompassing one was considered to be statistically significant. All statistical tests were two-sided.

## RESULTS

The characteristics of the patients are shown in Table 1. The median follow-up period was 40 (interquartile range [IQR]: 27–59) months and the median age was 62 (IQR 49–70) years. Of the 142 patients, 31 (22%) developed late rectal complications, including 24 (17%) with Grade 1 toxicity, 6 (4%) with Grade 2, and 1 (1%) with Grade 3. The median time from the start of radiotherapy to onset of late rectal complications was 15 (IQR 12–24) months.

**Table 1:** Patient characteristics (n = 142)

Characteristics		n	%
Age (y)	Median (range)	62 (29–93)	
Body mass index (kg/m^2^)	Median (range)	21.3 (14.9–36.9)	
Smoking	Brinkman index ≥200	35	25
Diabetes	Yes	6	4
Anticoagulant	Yes	8	6
FIGO (2008)	IB1-IB2	47	33
	IIA1-IIB	79	56
	IIIA-IIIB	16	11
Pelvic lymph node	Positive	47	33
Paraaortic node	Positive	10	7
Histology	SCC	123	87
	non-SCC	19	13
Chemotherapy	None	38	27
	Nedaplatin	77	54
	Cisplatin	10	7
	Paclitaxel and carboplatin	17	12
ICBT	4 times	64	45
	3 times	78	55

Abbreviations: FIGO = International Federation of Gynecology and Obstetrics, SCC = squamous cell carcinoma, ICBT = intracavitary brachytherapy

EDR_e_s for D_0.1cc_, D_2cc_ and D_5cc_ are shown in Table 2. The mean EQD_2_ values for D_0.1cc_, D_2cc_ and D_5cc_ are shown in Table 3. The mean source strength of Ir-192 (cGy.m^2^.h^−1^. ± SD) was 2.84 ± 0.58. Correlation coefficient analysis showed a strong correlation of EDR_e_ with source strength, and a moderate correlation of EDR_e_ with rectal dose, but no correlation of source strength with rectal dose (Supplementary Table S2). Differences in the mean EDR_p_ or EQD_2_ for patients with and without rectal complications (Grade 0 vs Grade ≥ 1) are shown in Table 3. Patients with rectal complications received a significantly higher mean EDR_p_ for D_0.1-5cc_ and had a greater EQD_2_ for D_0.1-5cc_. Parameters for D_2cc_ had the highest area under the ROC curve for both EDR_p_ (0.709) and EQD_2_ (0.672).

**Table 2:** Effective dose-rate in highest irradiation areas of the rectum for each session

DVH parameters	Mean (cGy/min)	SD	Maximum (cGy/min)	SD	Minimum (cGy/min)	SD
D_0.1cc_	142.80	52.30	184.52	72.58	115.20	40.44
D_2cc_	99.32	32.74	187.88	73.87	69.45	21.01
D_5cc_	83.02	26.17	185.83	72.37	54.99	16.14

Abbreviations: DVH = dose-volume histogram; SD = standard deviation

**Table 3:** Comparison of mean DVH parameters and dose rate to the rectum in patients with and without rectal complications

DVH parameters	Status	EQD_2_ (Gy)	SD	*P* value	Thres hold	AUC	EDR_p_ (cGy/min)	SD	*P* value	Thres hold	AUC
	Overall	88.35	12.72	0.018	83.20	0.64	142.80	52.30	0.002	125.61	0.68
D_0.1cc_	Complication (−)	87.13	12.82				135.14	46.60			
	Complication (+)	92.72	11.49				170.23	62.43			
	Overall	66.22	7.30	0.003	66.71	0.67	99.32	32.74	<0.001	98.20	0.71
D_2cc_	Complication (−)	65.35	7.32				94.13	30.16			
	Complication (+)	69.35	6.39				117.89	35.31			
	Overall	58.23	6.43	0.005	57.07	0.67	83.02	26.17	<0.001	81.52	0.71
D_5cc_	Complication (−)	57.46	6.44				78.88	24.44			
	Complication (+)	60.96	5.68				97.82	27.17			

Abbreviations: DVH = dose-volume histogram; EQD_2_ = equivalent dose in a 2-Gy fraction; EDR_p_ = effective dose rate for patients; SD = standard deviation; AUC = area under the curve

**Table 4:** Multivariate analysis (Cox proportional hazards model) for development of rectal complications

Variable	HR	95%CI	*p* value
Body mass index (kg/m^2^)	0.491	0.187–1.286	0.148
Smoker	0.778	0.333–1.816	0.561
EQD_2_ (D_2cc_)	2.585	1.020–6.550	0.045
EDR_p_ (D_2cc_)	3.378	1.492–7.647	0.004

Abbreviations; HR = hazard ratio; CI = confidence interval; EQD_2_ = equivalent dose in a 2-Gy fraction; EDR_p_ = effective dose rate for patients

A comparison of patients who did and did not develop late rectal complications showed no significant differences for age (<61 vs ≥61 years), body mass index (BMI) (<23.3 vs ≥23.3 kg/m^2^), heavy smoking (Brinkman index < 200 vs ≥200), histology (SCC vs non-SCC), concurrent chemotherapy (no vs yes), and standard loading of dwell positions (old vs new) (Supplementary Table S3). Among clinical parameters, source strength was significantly associated with mean EDR_p_ for D_2cc_ (p < 0.001) and the new standard loading of dwell positions showed a tendency to be related to the mean EDR_p_ for D_2cc_ (p = 0.058) (Supplement Table S4).

Multivariate analysis was performed using the mean EDR_p_ for D_2cc_, EQD_2_ for D_2cc_, heavy smoking and BMI. Heavy smoking and low BMI have been reported to be potential risk factors for late rectal complications [18]. Parameters for D_2cc_ were used in this analysis because they had the highest AUC values. Of these four parameters, EDR_p_ for D_2cc_ (HR: 3.38, p = 0.004) and EQD_2_ for D_2cc_ (HR: 2.59, p = 0.045) emerged as independent predictors for rectal complications (Table 4).

**Fig. 3.**
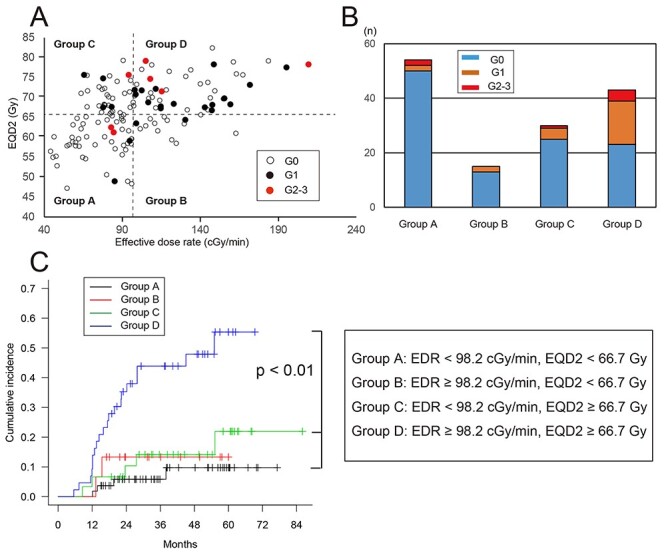
(a) Relationship of EDR_p_ for D_2cc_ and EQD_2_ for D_2cc_ for each patient. Four groups were established based on the cut-off threshold values in ROC analysis: Group A (low-EDR and low-EQD_2_), Group B (low-EDR and high-EQD_2_); Group C (high-EDR and low-EQD_2_), and Group D (high-EDR and high-EQD_2_); (b) Proportions of rectal complications in the four groups; (b) Actuarial rectal complication rates in the four groups.

Next, to investigate correlations of EQD_2_ for D_2cc_ and EDR_p_ for D_2cc_ with rectal complications, the patients were separated into four groups according to the cut-off values in ROC analyses: Group A (low-EDR and low-EQD_2_), Group B (low-EDR and high-EQD_2_), Group C (high-EDR and low-EQD_2_), and Group D (high-EDR and high-EQD_2_) (Fig. 3). There was a significantly higher rate of rectal complications in Group D compared to Groups A (p < 0.001) and C (p = 0.006), and a non-significant tendency for a higher rate in Group D compared to Group B (p = 0.055) (Fig. 3C).

## DISCUSSION

This study shows that the mean EDR_p_ in the highest irradiation area of the rectum is a significant independent predictor of late rectal complications in patients treated with HDR CT-based IGBT for cervical cancer. To our knowledge, this is the first report to show this relationship in a clinical setting. Previous studies of the relationship of the source strength of Ir-192 and late rectal complications have been inconclusive. We have found that the Ir-192 source strength affects late rectal complications after HDR-ICBT for cervical cancer [9, 10], but other studies suggest no relationship [11, 12]. These conflicting results might be explained by institutional variations in different fractionation schemes, types of applicators used, and dwell strategies. In the present study, the mean EDR_p_ in the highest irradiation area of the rectum changed due to standard loading of dwell positions, which implies that the EDR depends on the facility-specific standard dwell position. Therefore, we believe that the current study, in which we developed software for direct measurement of the EDR in the highest irradiation area of rectum, instead of measuring the Ir-192 source strength, provides new insights into late rectal complications after HDR-ICBT for cervical cancer.

It is clear that source strength affects EDR_p_ (Supplementary Table S4). In our center, an Ir-192 source is used with a strength of 370 GBq, equivalent to 4.08 cGy.m^2^.h^−1^. The half-life of Ir-192 is about 74 days and it is estimated that the agent needs about 30 days to decay to 2.84 cGy.m^2^.h^−1^ (the average source strength in this study). We usually exchange the source every three months (90 days). Therefore, we need to keep the rectal dose as low as possible during nearly one-third of the Ir-192 source lifetime. This could limit the treatment plan and become a problem in delivering a sufficient dose to the tumor during this treatment period. To avoid this, it may be necessary to use a lower source strength or to extend the source exchange period.

In this study, rectal EQD_2_ and mean EDR_p_ both affected late rectal complications after HDR-ICBT for cervical cancer. Many previous reports have found rectal EQD_2_ to be a robust predictor of late rectal complications. Therefore, we investigated how EQD_2_ and EDR_p_ affect these complications by establishment of four groups based on threshold levels for rectal EQD_2_ and EDR_p_. Patients with values above the respective thresholds had more rectal complications than other patients. It is of note that patients with EQD_2_ above its threshold did not have a higher frequency of rectal complications unless EDR_p_ also exceeded its threshold. Therefore, these findings suggest that either EQD_2_ or EDR_p_ need to be reduced in order to reduce rectal complications.

Many *in vitro* studies have shown that cell death depends on the dose rate, as well as the total dose. A dose-rate effect commonly occurs between 1 and 100 cGy/min and is not significant above or below that range [19]. In cervical cancer, the dose-rate effect has been analyzed in several LDR studies, which have shown that a higher dose rate is associated with a higher incidence of late complications [20, 21]. In contrast, the correlation between dose rate and late rectal complications in patients with cervical cancer treated with HDR-ICBT is uncertain. In fact, the dependence of cell death on dose rate in the HDR range is lower than that in the LDR range because the HDR range is minimally affected by sublethal damage repair.

There is also a marked difference in how radiation is applied in LDR and HDR brachytherapy. In LDR brachytherapy, all sources deliver the dose simultaneously at a constant dose rate at each point. Thus, each point irradiated at the same dose rate will receive the dose in the same total irradiation time. In contrast, in HDR brachytherapy, the delivery of radiation is more complex for the following reasons. First, the instant dose rate at each stepping point changes during the time-course of brachytherapy. Second, fractionations in HDR-ICBT compensate for the relative lack of protection of late-responding normal tissues. The effect of the dose rate in HDR brachytherapy has been examined in radiobiological models [22–24]. Using a single-plane template model, Manning et al. [22] postulated that cell killing is dependent on the dose rate, especially for cells in late responding tissues with short repair times. In contrast, Veigel et al. reported that dose-rate variation, rather than the dose-rate itself, causes cells to less efficiently activate a DNA damage response, compared to that after continuous irradiation [24]. However, in clinical use of HDR brachytherapy, the dose rate in the treatment volume has major variation temporally and statistically, as produced by the stepping source. Thus, events in actual treatment are likely to be much more complex than those in biological models. Therefore, additional experimental work is required to understand the biological background of the current study.

This study has several limitations. First, it had a retrospective design using data from a single institution, a low number of events, and a variety of chemotherapy regimens. Second, the threshold for rectal complications was set at Grade 1, which made it unclear if EDR is related to the severity of rectal complications. Moreover, which parameters in EDRs were important was also unclear. Within these limitations, we conclude that the mean EDR and EQD_2_ were associated with late rectal complications in patients treated with HDR CT-based IGBT for cervical cancer. A future prospective study with a larger sample size is needed to investigate how EQD_2_ and EDR influence late rectal complications.

## CONFLICT OF INTEREST

The authors report no conflict of interest.

## Supplementary Material

EDR_JRR_Sup_Rev_TableS1_final_rrab023

EDR_JRR_Sup_Rev_TableS2_final_rrab023

EDR_JRR_Sup_Rev_TableS3_final_rrab023

EDR_JRR_Sup_Rev2_TableS4_final_rrab023
