# Supplementary material for: Dose rate in the highest irradiation area of the rectum correlates with late rectal complications in patients treated with high-dose-rate computed tomography-based image-guided brachytherapy for cervical cancer
Source: J Radiat Res. 2021 Apr 19;62(3):494–501. doi: 10.1093/jrr/rrab023 (PMC8127676; doi:10.1093/jrr/rrab023)
Supplement: EDR_JRR_Sup_Rev_TableS1_final_rrab023 [file edr_jrr_sup_rev_tables1_final_rrab023.docx]

**Supplemental table S1.** Examples of radiation source arrangements used in our hospital

| **Cavity length** | **Old sequence (n=27)** | | | **New sequence** **(n=115)** | | |  |
| --- | --- | --- | --- | --- | --- | --- | --- |
|  |  | **Dwell position number** | **Weight** |  | **Dwell position number** | **Weight** |  |
| 7 cm | Ch 1: | 1・3・5・7 | 1.00 | Ch 1: | 1-7 * | 1.00 |  |
|  | Ch 2: | 1・3・5・7 | 1.00 | Ch 2: | 1-7 * | 1.00 |  |
|  | Ch 3: | 1・・4・・7・・・11・・・15・・・19・・・23 | 1.14 | Ch 3: | 1-23 * | 0.61 |  |
|  |  |  |  |  |  |  |  |
| 6 cm | Ch 1: | 1・3・5・7 | 1.00 | Ch 1: | 1-7 * | 1.00 |  |
|  | Ch 2: | 1・3・5・7 | 1.00 | Ch 2: | 1-7 * | 1.00 |  |
|  | Ch 3: | 1・・4・・7・・・11・・・15・・・19 | 1.14 | Ch 3: | 1-19 * | 0.63 |  |
|  |  |  |  |  |  |  |  |
| 5 cm | Ch 1: | 1・3・5・7 | 1.00 | Ch 1: | 1-7 * | 1.00 |  |
|  | Ch 2: | 1・3・5・7 | 1.00 | Ch 2: | 1-7 * | 1.00 |  |
|  | Ch 3: | 1・・4・・7・・・11・・・15 | 1.14 | Ch 3: | 1-15 * | 0.67 |  |
|  |  |  |  |  |  |  |  |
| 4 cm | Ch 1: | 1・3・5・7 | 1.00 | Ch 1: | 1-7 * | 1.00 |  |
|  | Ch 2: | 1・3・5・7 | 1.00 | Ch 2: | 1-7 * | 1.00 |  |
|  | Ch 3: | 1・・4・・7・・・11 | 1.14 | Ch 3: | 1-11 * | 0.73 |  |

* All dwell positions in the range were activated.
