# Supplementary material for: Dose rate in the highest irradiation area of the rectum correlates with late rectal complications in patients treated with high-dose-rate computed tomography-based image-guided brachytherapy for cervical cancer
Source: J Radiat Res. 2021 Apr 19;62(3):494–501. doi: 10.1093/jrr/rrab023 (PMC8127676; doi:10.1093/jrr/rrab023)
Supplement: EDR_JRR_Sup_Rev_TableS2_final_rrab023 [file edr_jrr_sup_rev_tables2_final_rrab023.docx]

**Supplemental Table S2.** Correlation analyses between source strength and EDR_e_, dose and EDR_e_, and dose and source strength of Ir-192 in each brachytherapy session

| **Comparison** | **DVH parameters** | **Correlation coefficient** | **95% CI** |
| --- | --- | --- | --- |
| Source strength vs. EDR_e_ | D0.1cc | 0.62 | 0.56 - 0.67 |
|  | D2cc | 0.71 | 0.66 - 0.75 |
|  | D5cc | 0.73 | 0.69 - 0.77 |
| Dose vs. EDR_e_ | D0.1cc | 0.63 | 0.58 - 0.68 |
|  | D2cc | 0.55 | 0.49 - 0.61 |
|  | D5cc | 0.51 | 0.44 - 0.57 |
| Dose vs. Source strength | D0.1cc | 0.11 | 0.02 - 0.19 |
|  | D2cc | 0.10 | 0.01 - 0.19 |
|  | D5cc | 0.09 | 0.00 - 0.18 |

Abbreviations: EDR_e_ = effective dose rate for each session; DVH = dose-volume histogram; CI = confidence interval
