# Supplementary material for: Dose rate in the highest irradiation area of the rectum correlates with late rectal complications in patients treated with high-dose-rate computed tomography-based image-guided brachytherapy for cervical cancer
Source: J Radiat Res. 2021 Apr 19;62(3):494–501. doi: 10.1093/jrr/rrab023 (PMC8127676; doi:10.1093/jrr/rrab023)
Supplement: EDR_JRR_Sup_Rev_TableS3_final_rrab023 [file edr_jrr_sup_rev_tables3_final_rrab023.docx]

**Supplemental Table S3.** Univariate analysis of development of late rectal complications

| **Variable** | **Grade 0** | **Grade ≥1** | ***p* value** |
| --- | --- | --- | --- |
| Age (y) |  |  |  |
| <61 | 52 | 12 | 0.541 |
| ≥61 | 59 | 19 |  |
| Body mass index (kg/m^2^) |  |  |  |
| <23.3 | 77 | 26 | 0.171 |
| ≥23.3 | 34 | 5 |  |
| Smoking (Brinkman index) |  |  |  |
| <200 | 83 | 28 | 1.000 |
| ≥200 | 24 | 7 |  |
| Histology |  |  |  |
| SCC | 97 | 26 | 0.565 |
| non-SCC | 14 | 5 |  |
| Chemotherapy |  |  |  |
| None | 43 | 12 | 1.000 |
| Yes | 68 | 19 |  |
| Dwell position |  |  |  |
| Old | 21 | 6 | 1.000 |
| New | 90 | 25 |  |

SCC = squamous cell carcinoma
