# Supplementary material for: Dose rate in the highest irradiation area of the rectum correlates with late rectal complications in patients treated with high-dose-rate computed tomography-based image-guided brachytherapy for cervical cancer
Source: J Radiat Res. 2021 Apr 19;62(3):494–501. doi: 10.1093/jrr/rrab023 (PMC8127676; doi:10.1093/jrr/rrab023)
Supplement: EDR_JRR_Sup_Rev2_TableS4_final_rrab023 [file edr_jrr_sup_rev2_tables4_final_rrab023.docx]

**Supplemental Table S4.** Univariate analysis of clinical parameters affecting mean EDR_p_ for D2cc

| **Variable** | **mean EDR_p_ for D_2cc_ (cGy/min)** | **SD** | ***p* value** |
| --- | --- | --- | --- |
| Age (y) |  |  |  |
| <61 | 96.06 | 34.01 | 0.210 |
| ≥61 | 101.99 | 31.63 |  |
| Body mass index (kg/m^2^) |  |  |  |
| <23.3 | 100.74 | 32.80 | 0.398 |
| ≥23.3 | 95.56 | 32.70 |  |
| Smoking (Brinkman index) |  |  |  |
| <200 | 97.00 | 31.01 | 0.141 |
| ≥200 | 106.42 | 37.14 |  |
| Source strength (cGy.m^2^.h^-1^) |  |  |  |
| <2.84 | 80.04 | 21.27 | <0.001 |
| ≥2.84 | 120.30 | 30.10 |  |
| Histology |  |  |  |
| SCC | 97.61 | 35.79 | 0.962 |
| non-SCC | 99.58 | 32.39 |  |
| Chemotherapy |  |  |  |
| None | 99.53 | 26.42 | 0.515 |
| Yes | 99.19 | 36.32 |  |
| Dwell position |  |  |  |
| Old | 87.88 | 27.86 | 0.058 |
| New | 102.01 | 33.33 |  |

SCC = squamous cell carcinoma; EDR_p_ = effective dose rate for patients
